# Orientation of the Electronic Qy Transition Dipole Moment in Chlorophyll a

**DOI:** 10.1021/acs.jpcb.6c01052

**Published:** 2026-05-23

**Authors:** Clark Zahn, Probal Nag, Till Stensitzki, Henrike Müller-Werkmeister, Igor Schapiro, Karsten Heyne

**Affiliations:** † Department of Physics, Free University Berlin, Arnimallee 14, D-14195 Berlin, Germany; ‡ Department of Physics, Technical University Dortmund, Otto-Hahn-Str. 4, D-44227 Dortmund, Germany; § Institute of Chemistry, University of Potsdam, Karl-Liebknecht-Str. 24-25, 14476 Potsdam, Germany; ∥ Institute of Chemistry, The Hebrew University of Jerusalem, Edmond J. Safra Campus, 9190401 Jerusalem, Israel

## Abstract

The orientation of the Q_*y*_ transition dipole moment (tdm) in Chlorophyll a (Chl a) is a fundamental parameter for optimizing energy transfer in light harvesting systems. Here, we apply polarization resolved transient VIS-pump/IR-probe and 2D-IR spectroscopy in combination with density functional theory (DFT) calculations to determining the orientation of the *Q*_*y*_ tdm for hexacoordinated Chl a. Analysis of the 2D-IR spectra provide a comprehensive study of the C=O vibrations, allowing us to refine the modeling of the vibrational tdm (vtdm) vectors. Polarization resolved transient IR spectra provide relative angles between the *Q*_*y*_ tdm and different probed vtdms, which determine cones of possible orientations of the *Q*_*y*_ tdm. Calculating the intersection of three cones from linearly independent vtdm vectors, we determine the orientation of the *Q*_*y*_ tdm. We find that the *Q*_*y*_ tdm for hexacoordinated Chl a is oriented along the *y*-axis of the macrocycle, exhibiting only a small angular deviation within −1° to 4°.

## Graphical abstract

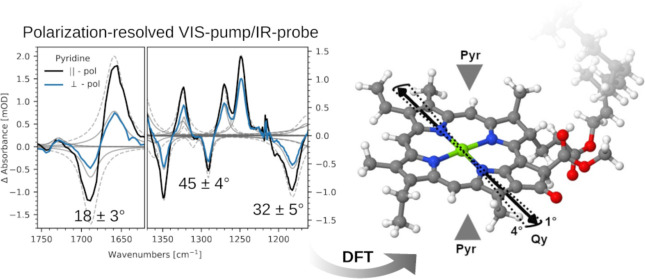

## Introduction

Chl a is widely known for its important role in photosynthesis. Its refined photophysical properties allow for a highly efficient and directional energy transfer, playing a crucial role in light harvesting.^1–3^ Chl a shares many properties with other cyclic tetrapyrroles, exhibiting two broad absorption bands, the Soret (B) band (350–450 nm) and the *Q*-band (550–680 nm), see Figure 1. Following the Gouterman Model,^4,5^ each band comprises two electronic states with distinct geometrical orientations, denoted *B*_*x*_, *B*_*y*_ and *Q*_*x*_, *Q*_*y*_, where the subscripts *x* and *y* indicate the orientations of the corresponding tdms within the plane of the macrocycle, see Figure 1 inset. In this regard, the orientation of the *Q*_*y*_ tdm is a key factor for enabling highly directional and efficient energy transfer.^1^ Thus, determining the precise orientation of the *Q*_*y*_ tdm within the molecular structure is crucial to fully understand energy transfer in the light harvesting complex. This led to different efforts investigating the orientation of the *Q*_*y*_ tdm.^6–9^ In the most recent report, Linke et al.^9^ combined DFT calculations with polarization resolved femtosecond visible pump-infrared probe spectroscopy to obtain the three-dimensional orientation of the *Q*_*y*_ tdm of Chl a in d_8_-toluene. For this method, the analysis critically depends on the assignment of normal modes and accurate modeling of the vtdms.

**Figure 1:**
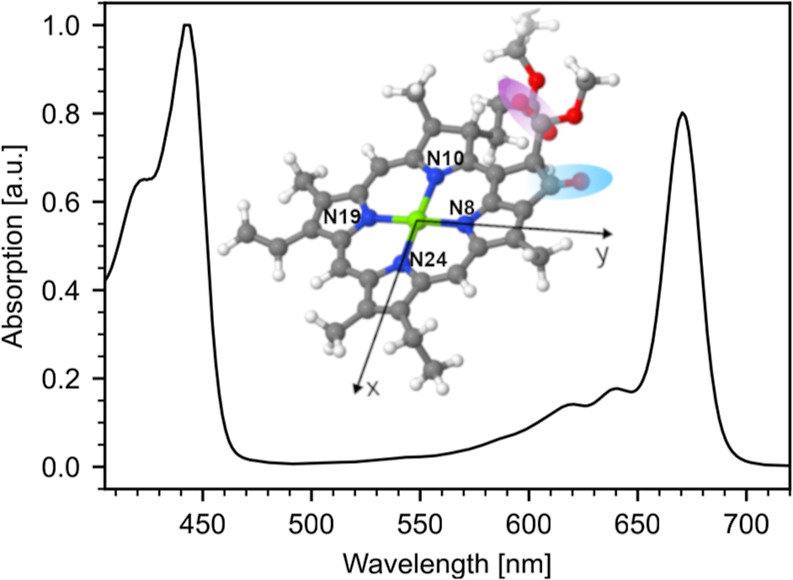
Normalized visible steady-state absorption spectrum of hexacoordinated Chl a in pyridine. Inset: Geometry optimized structure of Chl a macrocycle and its canonical *x*- and *y*-axis. Atoms are color coded: carbon (gray), nitrogen (blue), magnesium (green), oxygen (red), and hydrogen (white). The keto C=O and 10a-ester C=O groups are highlighted in light blue and light purple, respectively. The 7c-ester C=O group is hidden behind the acetate group.

In our recent study of hexacoordinated Chl a in pyridine,^10^ we found that the anisotropy of the carbonyl stretching vibration ν­(C=O)_1_ at 1698 cm^–1^ differs significantly compared to the previous report by Linke et al.^9^ Moreover, inspection of the other carbonyl stretching band ν­(C=O)_2_ at 1739 cm^–1^ showed that only the ν­(C=O)_1_ at 1698 cm^–1^ is sensitive to selective *Q*_*x*_ and *Q*_*y*_ excitation across the Q-band spectrum. This is particularly unexpected, as the DFT calculations modeled the two normal modes as coupled in-phase and out-of-phase vibrations of the keto C=O and 10a-ester-C=O groups.^9^ In the presented work, we employ polarization resolved transient IR-probe spectroscopy, together with polarization resolved femtosecond 2D-IR spectroscopy, allowing for an in-depth study of the vibrational signatures and resolving the origin of the carbonyl stretching modes ν­(C=O)_1_ and ν­(C=O)_2_. With these results, we refine the theoretical modeling and improve on previous work by Linke et al.,^9^ providing a clear determination of the orientation of the *Q*_*y*_ tdm in hexacoordinated Chl a. In contrast to previous reports investigating the orientation of the *Q*_*y*_ tdm,^6–9^ our results show that the *Q*_*y*_ tdm in hexacoordinated Chl a is oriented almost perfectly along the *y*-axis (N8–N19, Figure 1) of the macrocycle.

## Materials and Methods

### Sample Preparation

Chl a from spinach was purchased from Sigma-Aldrich. Samples were dissolved in pyridine and prepared in sample cells with a thickness of 100 μm, with a sample concentration of 1.5 mM-3 mM corresponding to an absorption of ∼2.5 OD at 671 nm.

### Transient Absorption

Femtosecond laser pulses are generated starting from Nd/YAG laser system (Pharos, Light Conversion) delivering 330 fs pulses at 1030 nm, operated at 1 kHz. Visible pump pulses are generated by a home-built two-stage stage NOPA with an output of ∼15 μJ. An acousto-optic programmable dispersive filter (AOPDF) (Dazzler, Fastlite) is used to compress and spectral filter the pump pulse yielding a (130 ± 20) fs pulse with a spectral width of FWHM (10 ± 3) nm. To ensure photoselection conditions, the pump-pulse is further attenuated to 0.2 μJ–0.5 μJ, exciting less than 20% of the sample. A λ/2-plate is used to control the polarization of the pump-beam, alternating between perpendicular and parallel pump - probe configuration. IR probe beams in the spectral range from 1100–1800 cm^–1^ (bandwidth of 200–300 cm^–1^) are generated by an optical parametric amplifier (Orpheus and Lyra, Light Conversion). Two reflections of the fs mid-IR pulse were taken, designated as signal and reference. Both beams pass through the same sample volume, with the reference pulse arriving 1.5 ns earlier. Both signal and reference are detected by dispersing the beams with an imaging spectrograph and recorded simultaneously recorded by a 128 × 128 pixel MCT-array (2DMCT, PhaseTech Spectroscopy, Inc.). The spectral resolution is better than 3 cm^–1^. The sample is moved with a Lissajous-scanner to ensure a fresh sample volume between consecutive pump pulses. The probe pulses are delayed using a mechanical translation stage. The system response duration was about (300 ± 50) fs.

### 2D-IR Spectroscopy

Femtosecond laser pulses are generated starting from a Ti/Sapphire laser system (Spitfire Ace PA, Spectra Physics) delivering 40 fs pulses at 800 nm, operated at 1 kHz. The IR beam is generated using a multistage optical parametric amplifier (HE-TOPAS, Light Conversion) pumped with 8 W. The amplifier provides 2 W–3 W of signal and idler output, which are mixed via difference-frequency generation (nDFG extension for TOPAS, Light Conversion) to produce IR pulses with energies up to 30 μJ centered at 1650 cm^–1^ (FWHM 500 cm^–1^). The resulting IR beam is directed into a 2D-IR spectrometer (2DQuickIR, PhaseTech Spectroscopy, Inc.), where it is split into pump (90%) and probe (10%) components. Pump pulses are shaped using a 4f-acousto-optic modulator (AOM, PhaseTech Spectroscopy, Inc.) to generate pulse pairs with variable delays of up to 5 ps. A 4-frame phase-cycling scheme is employed to suppress scattered light, and a rotating frame of 1600 cm^–1^ was used for the scanning pulse. A mid-IR polarizer and λ/2-plate is used to set polarization accordingly. After passing through the sample, the probe beam is split into two polarizations with a polarizer and is dispersed in a spectrometer and detected with a 128 × 128 pixel MCT-array (2DMCT, PhaseTech Spectroscopy, Inc.), providing an effective spectral resolution of 3 cm^–1^ along the probe axis.

### Data Analysis

Data analysis was performed in Python using the skultrafast^11,12^ package.

### Computational Methods

Ground state optimization of Chl a was performed using the B3LYP^13,14^ density functional in combination with the 6-31G­(d)^15,16^ basis set. The stable minimum was confirmed by performing a frequency analysis, which resulted in zero imaginary frequencies. The optimized structure was then utilized to determine the vibrational transition dipole vectors at the same level of theory. As the keto C=O of the macrocycle and 10a-ester C=O groups displayed coupled stretch vibrations, a relaxed scan of the acetate group dihedral, containing the 10a-ester C=O group was performed to estimate the barrier for rotation of the ester C=O group. All calculations were performed using the Gaussian 16 software package.^17^

## Results and Discussion

Normalized steady-state absorption of Chl a in pyridine is shown in Figure 1. The spectrum displays clear Soret-band absorption at 390 to 460 nm and Q-band absorption at 570 to 670 nm, with the *Q*_*y*(0–0)_ transition at 670 nm.^6,18–21^ In pyridine Chl a is hexacoordinated and adapts a flat macrocycle.^22–25^

To determine the spatial orientation of the *Q*_*y*_ tdm, we performed polarization resolved VIS-pump/IR-probe transient absorption spectroscopy probing the spectral ranges 1620 cm^–1^–1755 cm^–1^ and 1160 cm^–1^–1370 cm^–1^. After excitation the induced anisotropy decays through rotational diffusion with a time constant of τ ∼90 ps.^9^ On a time-scale between 2 to 15 ps the change in anisotropy is negligible within the S/N.^10^ Figure 2 shows the transient spectra upon excitation of the *Q*_*y*_ maximum 670 nm for averaged delay times between 2 ps–15 ps.

**Figure 2:**
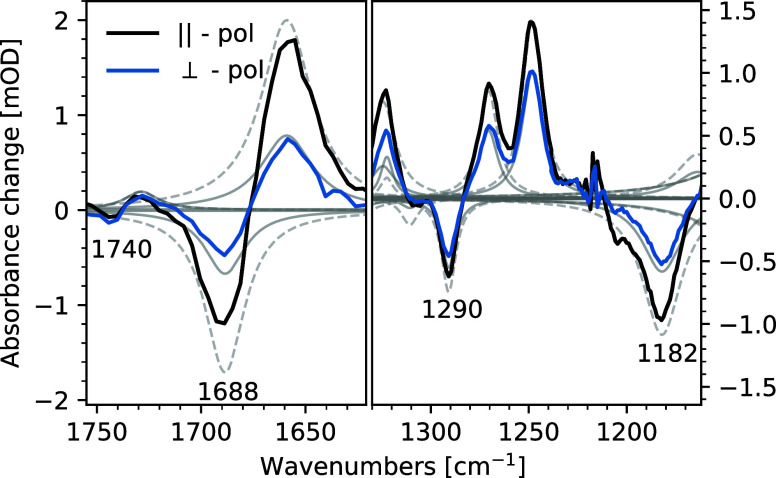
Polarization resolved transient infrared absorption spectra of hexacoordinated Chl a in pyridine, averaged over 2–15 ps. Visible excitation was at centered at the *Q*_*y*_ maximum at 67 nm. The spectral positions of the relevant bands are indicated by their corresponding wavenumbers. Gray lines represent fits to the experimental data using a sum of Lorentzian functions (parallel polarization: dashed lines; perpendicular polarization: solid lines).

Inspection of the higher frequency window from 1620 cm^–1^–1755 cm^–1^ shows positive and negative signal contributions attributed to the ester and keto carbonyl groups. Chl a has two ester C=O groups with overlapping frequencies at around 1740 cm^–1^, the 7c-ester C=O at the phytyl tail and the 10a-ester C=O at the acetate group. Yet, in VIS-pump/IR-probe experiment only the 10a-ester C=O is expected to show significant changes upon photoexciation.^26^ Hence, we assign the band at around 1740 cm^–1^ to the 10a-ester C=O stretching vibration. The ground-state bleach (GSB) located at 1690 cm^–1^ is assigned to the remaining C=O group, the keto C=O. For both, the 10a-ester and the keto C=O band, associated red-shifted positive excited state absorption (ESA) features are observed. The lower frequency window 1160–1370 cm^–1^ contains a complex manifold of vibrations typically associated with different combinations of C–N stretching, C–C stretching, and C–H in/out-of-plane deformation modes.^27^ In this context, the two bleaching bands at 1185 cm^–1^ and 1290 cm^–1^ are of particular interest, since they can be assigned to two distinct normal modes in the DFT calculations.

### Coupling of the Keto C=O and 10a-Ester C=O

Despite an apparently clear experimental assignment, theoretical modeling using DFT calculation identifies the two C=O bands at 1690 cm^–1^ and 1740 cm^–1^ as coupled and delocalized in-phase and out-of-phase stretching vibrations of the keto C=O and 10a-ester C=O.^9^ Since this cannot be ruled out from previous experiments, we investigated possible coupling of the keto C=O with the 10a-ester C=O by performing complementary polarization resolved 2D-IR spectroscopy measurements of Chl a in the spectral range 1665–1740 cm^–1^ (pump)/1650 to 1750 cm^–1^ (probe). As the IR absorption of pyridine obscures the 2D-IR signal in the spectral window of interest, we performed the 2D-IR measurements in *d*_8_-toluene. However, in *d*_8_-toluene Chl a is pentacoordinated and may form Dimers even at low concentrations.^18^ Yet, concentration dependent Vis pump/IR probe measurements of Chl a in *d*_8_-toluene do not show any significant change in the relative angle of the keto C=O band (see Supporting Information). Thus, it is highly unlikely that for the given concentrations, possible dimerization effects are affecting the coupling of the keto C=O and 10a-ester C=O. Therefore, while the change in coordination is expected to influence the vibrational and electronic structure, it is not expected to affect the coupling of the keto C=O and 10a ester-C=O.

Figure 3a shows a color-coded contour map of the isotropic 2D spectrum of Chl a in *d*_8_-toluene recorded at a waiting time of 500 fs. Blue color indicates negative signals associated with GSB, while red colors indicate positive signals attributed to ESA. The 2D-IR map exhibits characteristic diagonal features associated with the ester and keto C=O bands. In contrast to VIS-pump/IR-probe transient absorption, the 7c-ester C=O also contributes to the signal at 1715|1736 cm^–1^ and 1736|1736 cm^–1^. However, since the distance between the 7c-ester C=O and the keto C=O is quite large, we can neglect coupling between these groups. In the off-diagonal region we only find very weak (<10% of the diagonal peak, see signal strength Figure 3b) cross-peak contributions at 1693|1736 cm^–1^ and 1733|1678 cm^–1^, indicating limited vibrational coupling between the 1733 cm^–1^ and the 1693 cm^–1^ bands. Transient absorption change traces for parallel (circles) and perpendicular (squares) polarizations for the cross-peak contributions as well as the GSB at 1693| 1693 cm^–1^ are shown in Figure 3b. Analyzing the dichroic ratio D = *A*_∥_/*A*_⊥_ between the parallel and perpendicular signals, we calculated the relative orientation between the excited and probed vtdms as follows

1 $$\theta =\arccos\left(\sqrt{\left(2D-1\right)/\left(D+2\right)}\right)$$

**Figure 3:**
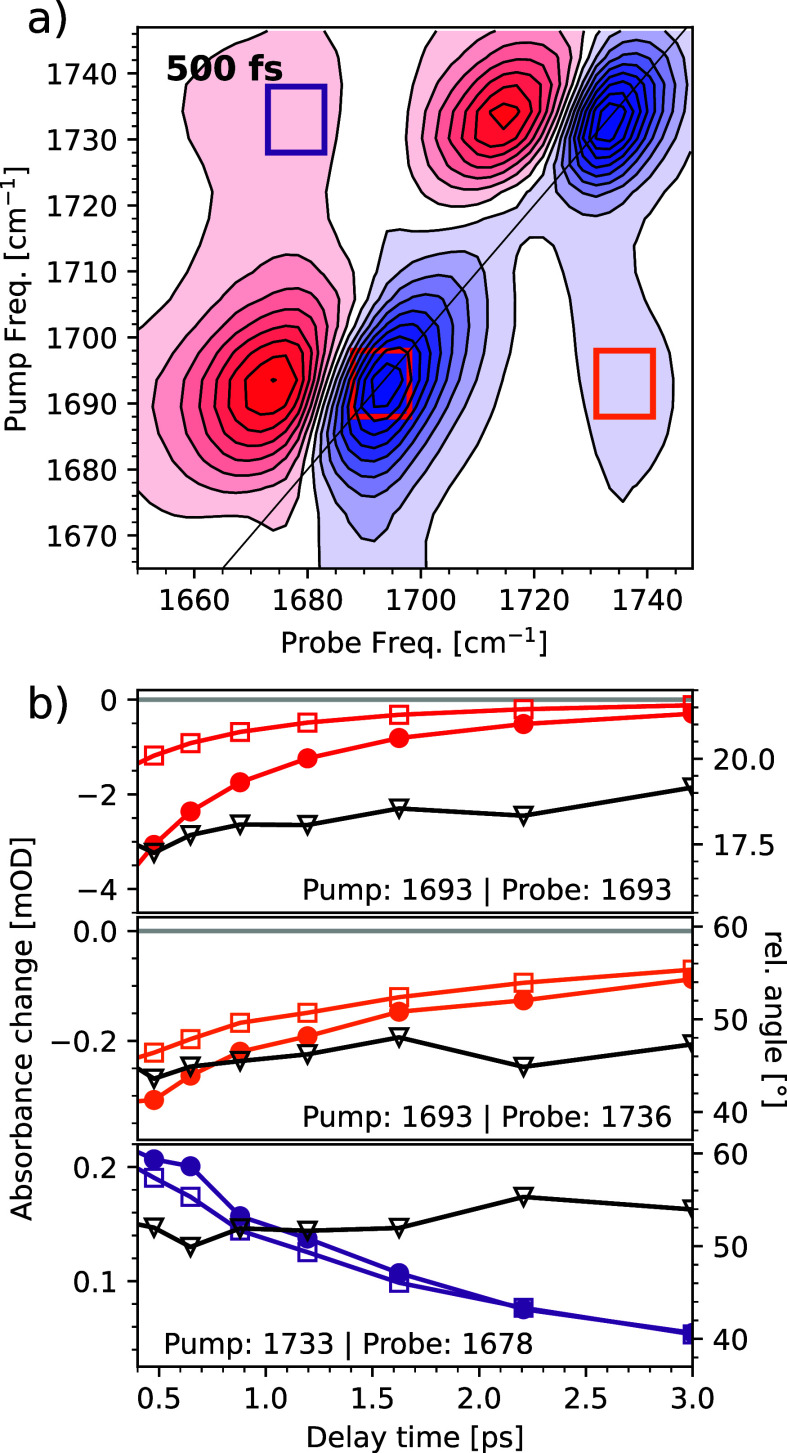
(a) Color coded map showing the isotropic 2D-IR spectrum of Chl a in *d*_8_-toluene recorded at a waiting time of 500 fs, for a pump range of 1665–1740 cm^–1^ and a probe range of 1630–1750 cm^–1^. Red color indicates positive signals while blue colors indicate negative signals; (b) Transient absorption change traces for selected diagonal and cross-peak positions as indicated in (a). Circles and squares indicate parallel and perpendicular probe polarization, respectively. Calculated relative angle traces are shown in black (triangles) with the corresponding *y*-axis displayed on the right.

Corresponding calculated transient relative angle traces for the selected wavenumbers are shown in Figure 3b, black triangles. Inspection of the relative angles of the off-diagonal cross-peaks yields angles close to a magic angle 54.7°. This is in agreement with an isotropic orientation distribution of the 10a-ester C=O group. From this we deduce that the acetate group, containing the 10a-ester C=O, can rotate around the connecting C–C bond, limiting possible coupling configurations with the keto C=O. The interpretation is further supported by femtosecond anisotropy excitation spectroscopy, tracing the relative angle of the keto C=O and 10a-ester C=O as a function of excitation wavelength (see Supporting Information). We find that the relative angle of the 1693 cm^–1^ band shows a clear dependence on the excitation wavelength dependence, while the 1733 cm^–1^ shows a magic angle (54.7°) configuration across all excitation wavelengths. Particularly, the magic angle configuration of the 1740 cm^–1^ band confirms the conformational flexibility of the acetate group. This is also in good agreement with a relaxed scan of the acetate dihedral, showing that the energy barrier for full 180° rotation of the acetate group is below 800 cm^–1^ (see Supporting Information Figure S2). At room temperature, this allows for significant conformational flexibility, which prevents consistent dipole alignment and limits the occurrence of vibrational coupling. Thus, we conclude that the C=O bands are more accurately described by localized C=O vibrations of the keto C=O and 10a-ester C=O, rather than delocalized in-phase and out-of-phase coupled modes. We assign the low energetic band at 1695 cm^–1^ to the keto C=O and the other band at 1735 cm^–1^ to the 10a-ester C=O. To decouple the vibrational modes of the keto C=O and 10a-ester C=O groups in the DFT calculations, we introduced an isotopic perturbation by substituting the oxygen of the 10a-ester with the heavier isotope ^18^O. The isotopic substitution breaks the energetic degeneracy and decouples two groups. The refined simulations yield a more accurate representation of the C=O vtdms, where the keto C=O vibration is strongly localized to its C=O group, with its vtdm mainly orientated along of the C=O bond.

### Orientation of the *Q*_*y*_ tdm

In order to determined the spatial orientation of the *Q*_*y*_ tdm from our data, we quantitatively analyze the anisotropy. We fit the spectra for both polarization directions using a sum of Lorentzian peaks. The overall fits are represented by the gray lines in Figure 2 (parallel polarization: dashed lines; perpendicular polarization: solid lines). From the fitted amplitudes for parallel and perpendicular polarization we can calculated the relative angles θ_*i*_ between the excited *Q*_*y*_ tdm and the probed vtdms, see eq 1. The experimental relative angles θ_*i*_ are summarized in Table 1. Errors are calculated using exhaustive search analysis.^28,29^ Matching the experimentally obtained relative angles θ_*i*_ with the orientation of the vtdms vectors from the isotope labeled ^18^O DFT calculations, we can determine the three-dimensional orientation of *Q*_*y*_ tdm.^9,28^ Taking into account the uncertainty ranges for θ_*i*_, we determine probability cones of possible orientations of the *Q*_*y*_ tdm for all three modes.

**Table 1:** Relative Angles θ_*i*_ with Uncertainty Ranges One Standard Deviation, ± 1σ between the *Q*_*y*_ tdm and the vtdms (Bleaching Signals in Figure 2) for Hexacoordinated Chl a in Pyrridine

mode	pyridine (exp)
ν(1185 cm^–1^)	(32 ± 5)°
ν(1290 cm^–1^)	(45 ± 4)°
ν(1690 cm^–1^)	(18 ± 3)°

For this, each relative angle was modeled by a Gaussian probability distribution centered at the best-fit value and a width equal to the 1σ error of the fit. Subsequently, the orientation of the *Q*_*y*_ tdm is determined by the intersection of the cones. The intersection is determined by multiplying the individual distributions, yielding the intersection area with a given probability distribution, indicating the most probable orientation. The color coded intersection area for the *Q*_*y*_ tdm orientation is displayed in Figure 4a. The probability decreases from red to blue to yellow.

**Figure 4:**
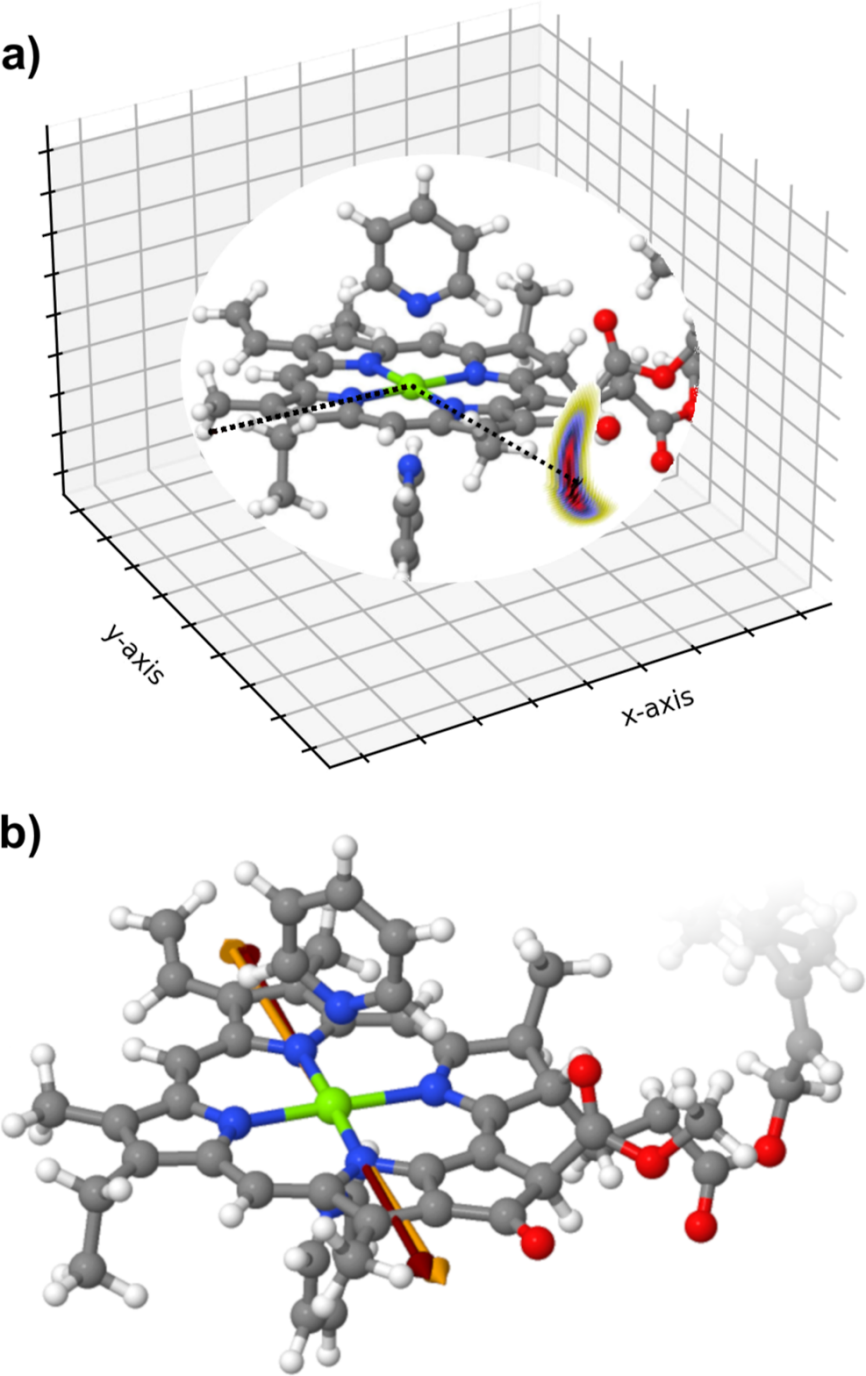
(a) Color coded area showing the probability for the 3D solution of the *Q*_*y*_ orientation on a unit sphere. The area is calculated by the intersection of all three relative angle ranges θ_*i*_ ± 1σ between the excited *Q*_*y*_ tdm and the vtdms. Chl a (scaled down) with associated *x* and *y* axis (black dotted lines) illustrates the orientation of the *Q*_*y*_ tdm with respect to the macrocycle plane; (b) Orientations of the *Q*_*y*_ tdm from DFT calculations (orange arrow) and from the best fit value from experiment (red arrow).

We can further narrow down the possible orientation by considering that for hexacoordinated Chl a the macrocycle adapts a flat configuration. Thus, it is very unlikely that the *Q*_*y*_ tdm points out of the *x*–*y* plane. Taking this into account, we find that the *Q*_*y*_ tdm in hexacoordinated Chl a is oriented almost perfectly along the *y*-axis of the macrocycle frame, exhibiting only a small angular deviation within the range of 4° to −1° (in direction away from the keto C=O). Comparison of the experimental results with DFT calculations of the *Q*_*y*_ tdm shows an excellent agreement between theory and experiment for hexacoordinated Chl a. The calculated *Q*_*y*_ tdm forms an angle of −2° with the *y*-axis and −1.4° with the *z*-axis, indicating that from the calculations the *Q*_*y*_ tdm is slightly tilted out of the macrocycle plane. The corresponding theoretical and experimental orientation of the *Q*_*y*_ tdm is shown in Figure 4b.

## Reevaluating the Orientation of the *Q*_*y*_ tdm

This result is significantly closer to the *y*-axis than previously reported. Early studies from the eighties and nineties of Chl a orientated in a film^6–8^ report an angle of ∼20° between the *Q*_*y*_ tdm and the *y*-axis. However, for these studies we expect that the influence of embedding Chl a in thin films strongly influence the coordination, which can directly influence the orientation of the electronic tdms.

More notably, a previous study by Linke et al.^9^ used the same approach as reported in this work, combining femtosecond polarization resolved VIS pump-IR probe spectroscopy with DFT calculations. However, Linke et al. reported a relative angle of (12 ± 3)° between the *Q*_*y*_ tdm and the *y*-axis. In reevaluating the results of Linke et al. we find three shortcomings of the previous analysis, which are addressed in the presented work.

A first issue arises from the modeling of the carbonyl stretching modes ν­(C=O)_1_(1698 cm^–1^) and ν­(C=O)_2_(1739 cm^–1^). Here, the two bands were modeled as respective normal modes of coupled in-phase and out-of-phase vibrations of the keto C=O and 10a-ester C=O. Yet, our 2D-IR data show that the two carboxyl bands are more accurately described by localized C=O vibrations with negligible coupling of the keto C=O and 10a-ester C=O. Our new data show that only the keto C=O at 1698 cm^–1^ shows meaningful anisotropy, while the rotational freedom of the ester C=O at 1739 cm^–1^ leads to an isotropic distribution, making the anisotropy of the band at 1739 cm^–1^ inconclusive. To address this issue, we substituted the oxygen of the 10a-ester C=O with the heavier isotope ^18^O and decouple the vibrational modes of the keto C=O and 10a-ester C=O groups in our DFT calculations. This breaks the vibrational degeneracy and coupling, yielding a more accurate representation of the C=O tdms. The second issue is that Linke et al. did not address possible solvent and coordination effects for Chl a in *d*_8_-toluene. In pentacoordinated Chl a the structural changes induce a displacement of the positively charged Mg^2+^, which directly affects the orientation of the electronic *Q*_*y*_ tdm. In addition, possible changes to the macrocycle, inducing variations to the normal modes and the orientation of the vtdms, are not accounted for in the DFT calculations.

Last, reevaluating the band assignment, we find that the vtdm vector associated with ν­(1290 cm^–1^) was attributed to a different vibration of similar frequency. However, using a more plausible scaling factor based on the spectral position of the GSB at 1185 cm^–1^ and 1290 cm^–1^, our assignment demonstrates a better agreement with the experimental data (see Supporting Information).

## Conclusion

In combining polarization resolved transient femtosecond IR spectroscopy with polarization resolved femtosecond 2D-IR spectroscopy we present a comprehensive in-depth study of the vibrational signatures of hexacoordinated Chl a. Analysis of the 2D-IR data reveals very weak (<10%) vibrational coupling between the 1733 cm^–1^ and the 1693 cm^–1^ bands. This challenges previous DFT calculations,^9^ which identified the two bands at 1693 cm^–1^ and 1733 cm^–1^ as coupled, delocalized in-phase and out-of-phase stretching vibrations of the keto C=O and 10a-ester C=O. Inspection of the relative angles of the 2D-IR cross-peaks suggests, that the vibrational coupling is limited by rotational freedom of acetate group, which includes the 10a-ester C=O. This is supported by relaxed scan of the acetate dihedral, showing that the energy barrier for rotation of the 10a-ester C=O group is below 800 cm^–1^. Thus, we conclude that the two C=O bands are more accurately described by localized C=O vibrations of the keto C=O (1695 cm^–1^) and 10a-ester C=O (1735 cm^–1^). By introducing an isotopic perturbation, substituting the oxygen of the 10a-ester with the heavier isotope ^18^O, we decouple the two C=O bands, allowing for a more accurate representation of the keto C=O vtdm vector. With this, we are able to determine the three-dimensional orientation of the *Q*_*y*_ tdm, showing that the *Q*_*y*_ tdm in hexacoordinated Chl a is oriented almost perfectly along the *y*-axis, exhibiting only a small angular deviation within 4° to −1° (away from the keto C=O). Comparison with DFT calculations of the *Q*_*y*_ tdm show an excellent agreement between theory and experiment for hexacoordinated Chl a. The presented results are significantly closer to the *y*-axis than reported by previous studies.^6–9^ However, previous studies from the eighties and nineties did not address the coordination of Chl a,^6–8^ while the shortcoming of the recent work by Linke et al. are addressed in this work.

## Supplementary Material



## Notes

Additional experimental data: Anisotropy excitation spectroscopy of the keto C=O and 10a-ester C=O; Possible dimerization effects of Chl a in d8-toluene affecting the coupling of the keto C=O and 10a-ester C=O; DFT calculation: Scan of the dihedral of the acetate group and detailed discussion on the assignment of the 1290 cm^–1^ band (PDF)
