# Supplementary material for: Orientation of the Electronic Qy Transition Dipole Moment in Chlorophyll a
Source: J Phys Chem B. 2026 May 23;130(22):5488–94. doi: 10.1021/acs.jpcb.6c01052 (PMC13244453; doi:10.1021/acs.jpcb.6c01052)
Supplement: Supplementary file 1 [file jp6c01052_si_001.pdf]

Supporting Information: Orientation of the electronic Qy Transition Dipole Moment in Chlorophyll a

Clark Zahn,[*,†] Probal Nag ,[‡] Till Stensitzki,[¶] Henrike Müller-Werkmeister,[¶] Igor Schapiro ,[‡,§] and Karsten Heyne[*,†]

†*Department of Physics, Free University Berlin, Arnimallee 14, D-14195 Berlin, Germany*

‡*Department of Physics, Technical University Dortmund, Otto-Hahn-Str. 4, D-44227 Dortmund, Germany*

¶*Institute of Chemistry, Physical Chemistry, University of Potsdam, Karl-Liebknecht-Str. 24-25, 14476 Potsdam, Germany*

§*Institute of Chemistry, The Hebrew University of Jerusalem, Edmond J. Safra Campus, 9190401 Jerusalem, Israel*

E-mail: clark.zahn@fu-berlin.de; karsten.heyne@physik.fu-berlin.de

Phone: +49 30 838 56107

Anisotropy excitation spectroscopy of the keto C=O and 10a-ester C=O

Anisotropy excitation spectra of Chl a in d_8-toluene upon excitations between $570\,\mathrm{nm}$ and $660\,\mathrm{nm}$ for the keto C=O ($1688\,\mathrm{cm}^{-1}$) and 10a-ester C=O ($1740\,\mathrm{cm}^{-1}$) are shown in Figure

S1. Inspection of the spectra shows that while the keto C=O exhibits a clear dependence on the excitation wavelength, the 10a-ester C=O remains invariant, showing a magic angle (54.7°) configuration across all excitation wavelengths. As we expect the Q_x and Q_y transition dipole moments (tdm) to be close to orthogonal, such a behavior contradicts the picture of coupled C=O vibrations. Moreover, particularly the magic angle configuration of the $1740\,\mathrm{cm}^{-1}$ band suggests a large conformational flexibility of the acetate group. This rotation of the acetate group prevents consistent dipole alignment and hinders the occurrence of vibrational coupling.

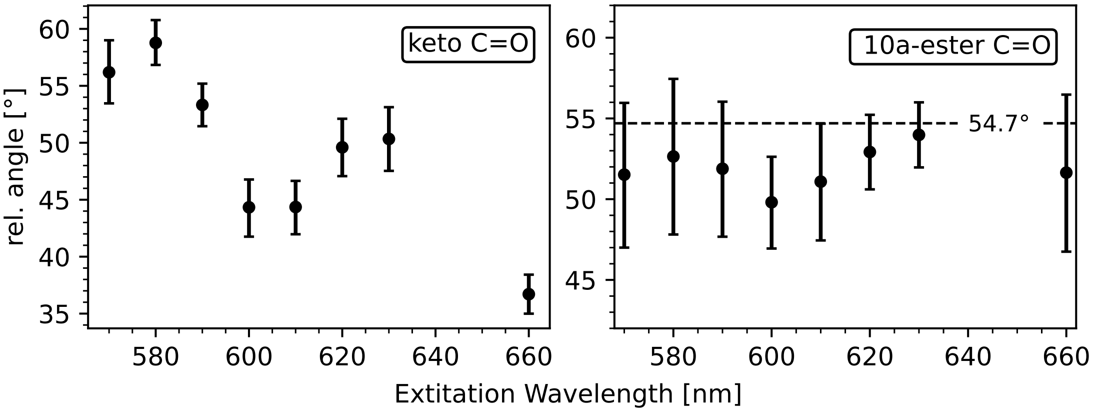

Figure S1 Relative angles for different excitation wavelengths of the keto C=O ($1688\,\mathrm{cm}^{-1}$) and 10a-ester C=O ($1740\,\mathrm{cm}^{-1}$) band of Chl a in d_8-toluene. The relative angle of the keto C=O shows a clear wavelengths dependence mirroring changing Qx and Qy excitation. In contrast the 10a-ester C=O d is invariant under Qx and Qy excitation.

Possible dimerization effects of Chl a in d_8-toluene affecting the coupling of the keto C=O and 10a-ester C=O

We instigated the concentration dependence of the relative angle of the keto C=O for Chl a in d_8-toluene, performing Vis pump/IR probe measurements for different concentrations. Using the same sample cell for all measurements, the change in concentration is identical to the change in absorbance of the Q band maximum at $660\,\mathrm{nm}$. Figure S2 shows the

relative angle of the keto C=O for different concentrations. Inspection of the relative angle shows that within the investigated range from $0.77\,\mathrm{OD}$ to $1.9\,\mathrm{OD}$, no significant change in the relative angle of the keto C=O was observed. Thus, we conclude that any dimerization effects are negligible within the error margin of the relative angle. Therefore, it is highly unlikely that any dimerization effects of Chl a in d_8-toluene affect the coupling of the keto C=O and 10a-ester C=O, as this would be directly visible in a change in the relative angle for an increased concentration.

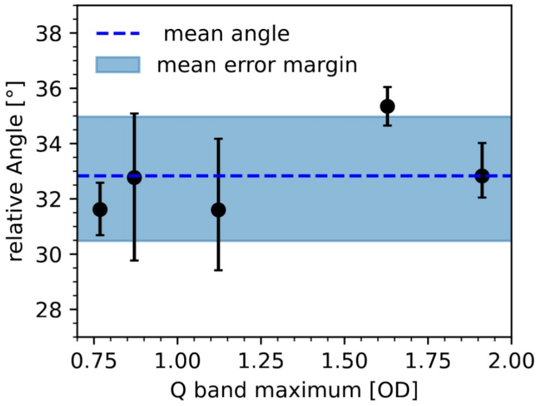

Figure S2 Relative angle of the keto C=O vtdm as a function of the Chl a Q band absorption, following excitation at $660\,\mathrm{nm}$. The blue area gives the 2σ error margin for the mean relative angle.

Scan of the dihedral of the acetate group

In order to investigate the conformational flexibility of the 10a-ester C=O and possible coupling between the keto C=O and 10a-ester C=O we performed a relaxed scan of the acetate group dihedral, containing the 10a-ester C=O group. The results show that the energy barrier for rotation of the 10a-ester C=O group is very low, see Figure S3. Thus, at room temperature, the 10a-ester C=O has significant conformational flexibility, leading to a wide distribution of accessible conformations between the keto C=O and 10a-ester C=O. This prevents consistent dipole alignment, in line with the experimentally observed isotropic distribution of possible configurations.

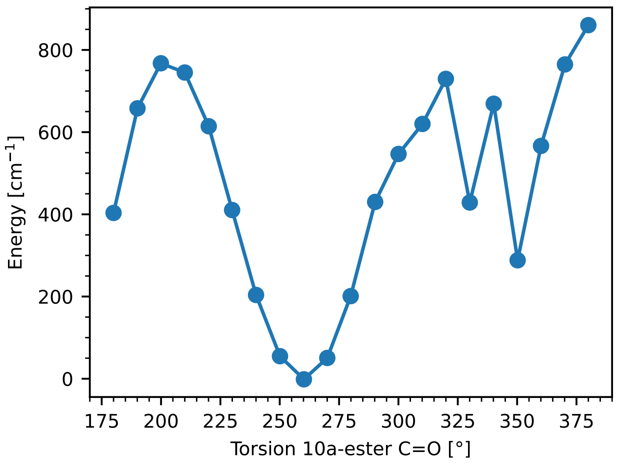

Figure S3 Energy barrier in cm^{-1} for rotation of the 10a-ester C=O group calculated from DFT.

Assignment of the 1290 cm^{-1} band

In the work published by Linke et al.[S1] they assigned the band at $1290\,\mathrm{cm}^{-1}$ to the normal mode 251, see Table S1. In reevaluating the band assignment, we have assigned this band to the mode 239. A reason for this might be using a different scaling factor. While Linke et al. did not provide any scaling factor, small differences in the scaling vector shift the frequency of modes significantly. However, it seems very likely that Linke et al. determined the scaling factor by matching the theoretical and experimental positions of the C=O bands at $1700\,\mathrm{cm}^{-1}$ and $1740\,\mathrm{cm}^{-1}$, yielding a factor of 0.94, which explains the assignment of the $1290\,\mathrm{cm}^{-1}$ band. However, the B3LYP functional typically overestimates higher frequency modes, such as C=O vibrations.[S2] On the other hand, considering the bleaching bands at $1185\,\mathrm{cm}^{-1}$ and $1290\,\mathrm{cm}^{-1}$ a scaling factor of 0.967 seems more suitable, matching the experimental spectrum, see Figure S4.

Table S1 Vibrational normal modes in the range $1200\,\mathrm{cm}^{-1}$ - $1375\,\mathrm{cm}^{-1}$ with an IR intensity > 50 obtained from DFT calculations by Linke et al.[S1] Gray: ν(C=C)($1288\,\mathrm{cm}^{-1}$) assignment by Linke et al. Green: more plausible assignment using a different scaling factor; *Scaling factor: 0.967; **Scaling factor: 0.94.

Mode No.	Freq [cm^{-1}]	scaled* Freq [cm^{-1}]	scaled** Freq [cm^{-1}]
225	1246	1205	1171
239	1329	1285	1249
247	1354	1309	1272
251	1374	1328	1291
264	1419	1372	1334

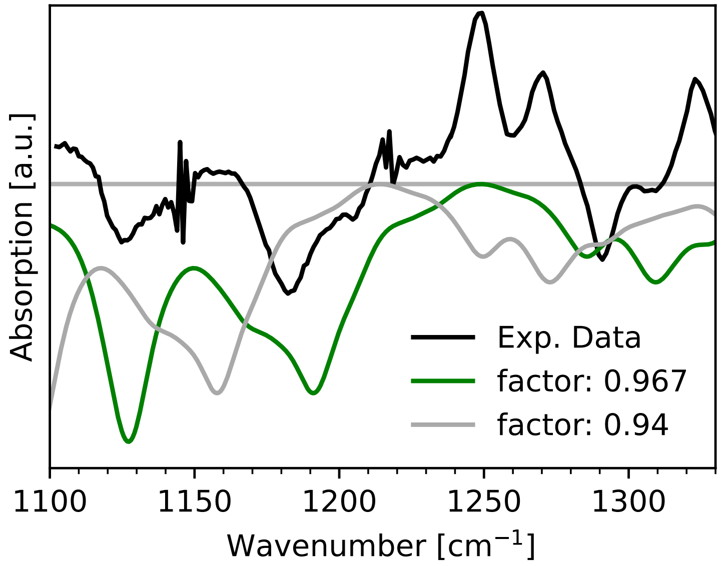

Figure S4 Comparison of experimental (black) and theoretical spectra with a scaling factor of 0.967 (green) and 0.94 (gray). Both theoretical spectra are simulated modeling the frequency from each mode with a Lorentzian peak with a width of $10\,\mathrm{cm}^{-1}$ and an amplitude representing the calculated IR intensity. The theoretical spectrum utilizing a scaling factor of of 0.967 demonstrates better agreement with the experimental peak positions at $1185\,\mathrm{cm}^{-1}$ and $1290\,\mathrm{cm}^{-1}$.

References

(S1) Linke, M.; Lauer, A.; von Haimberger, T.; Zacarias, A.; Heyne, K. Three-Dimensional Orientation of the Qy Electronic Transition Dipole Moment within the Chlorophyll

a Molecule Determined by Femtosecond Polarization Resolved VIS Pump-IR Probe Spectroscopy. *J. Am. Chem. Soc* **2008**, *130*, 14904–14905.

(S2) Laury, M. L.; Carlson, M. J.; Wilson, A. K. Vibrational frequency scale factors for density functional theory and the polarization consistent basis sets. *Journal of Computational Chemistry* **2012**, *33*, 2380–2387.
